# Reasons for using indoor tanning devices: A systematic review of qualitative evidence

**DOI:** 10.1111/bjhp.12610

**Published:** 2022-07-09

**Authors:** Martin Eden, Stephanie Lyons, Paul Lorigan, Katherine Payne, Adele C. Green, Tracy Epton

**Affiliations:** ^1^ Manchester Centre for Health Economics The University of Manchester Manchester UK; ^2^ Manchester Centre for Health Psychology The University of Manchester Manchester UK; ^3^ Cancer Research UK Manchester Institute The University of Manchester Manchester UK; ^4^ Christie NHS Foundation Trust Manchester UK; ^5^ QIMR Berghofer Medical Research Institute Brisbane Australia

**Keywords:** indoor tanning devices, melanoma, qualitative studies, skin cancer, systematic review

## Abstract

**Purpose:**

Despite the established causal links to skin cancer, skin ageing and eye inflammation, people continue to use indoor tanning devices (hereafter ‘sunbeds’). Understanding the reasons underlying the use of sunbeds is essential for developing effective interventions. The purpose of this study was to collate all existing evidence from qualitative papers published to date that had assessed motivations for using sunbeds.

**Methods:**

Six databases were searched from inception to February 2020 for qualitative studies that explored adults' experiences of using sunbeds. Sixteen studies met the inclusion criteria, and a narrative evidence synthesis was used to collate findings from each primary study.

**Results:**

Users of sunbeds were motivated primarily by aesthetic concerns but also by perceived psychological benefits (well‐being, confidence and ‘fitting in’) and physical benefits (improvement in skin conditions such as acne, acquiring vitamin D and preventing sunburn). People also chose indoor tanning over alternatives such as fake tans because they considered the alternatives unacceptable and did not consider indoor tanning a serious health risk. To date, no studies have explored alternatives to meeting non‐aesthetic needs related to the use of sunbeds.

**Conclusions:**

This comprehensive explanation for the practice of indoor tanning provides the basis for development of complex interventions to reduce the harm caused by using sunbeds. Effective interventions should include promotion of alternatives, such as different methods of relaxing, to satisfy underlying motivations, changing social norms and correcting misperceptions about health benefits.

## BACKGROUND

The use of indoor tanning devices (sunbeds) increases the risks of all major skin cancers (International Research Agency on Cancer [IRAC], 2012). Specifically, indoor tanning increases the risk of keratinocyte cancers by up to 67% (Wehner et al., 2012) and the risk of melanoma by 20% overall, and by 59% when first use occurs before age 35 (Boniol et al., 2012). In 2009, indoor tanning devices were classified as Class 1 carcinogens by the WHO (IRAC, 2012). Additionally, sunburns, skin ageing, eye inflammation and temporary immunosuppression are all associated with indoor tanning devices (WHO, 2017).

Indoor tanning is regulated through a range of measures, that is, licensing commercial indoor tanning devices, regulating use amongst high‐risk subgroups (those under 18 years; those with fair skin), controlling exposure, implementing taxes and mandatory notification of the risks (WHO, 2017). Australia, Brazil and Iran have implemented outright bans (Rodriguez‐Acevedo et al., 2019). However, despite the 2009 carcinogen classification and the above regulations, people still choose to tan indoors. A recent systematic review and meta‐analysis revealed that, between 2013 and 2018, more than 40,000 adults across Europe, North America and Australia reported the previous use of tanning devices (Rodriguez‐Acevedo et al., 2019). Similarly, it is estimated roughly 36% of White adults across North America have used indoor tanning devices (Rodriguez‐Acevedo et al., 2019).

A recent study found a public health campaign and legislation to ban sunbeds in England would save lives and be an effective use of the health care budget (Eden et al., 2022). This suggests that policy‐based initiatives delivered with an intervention to change behaviour can substantially decrease sunbed use. Understanding the reasons why people indoor‐tan is crucial to determine the focus of behavioural change interventions, and the behavioural change techniques (BCTs) to reduce use (Michie et al., 2011; Stapleton et al., 2010). A recent narrative review, largely of quantitative survey data, reported that the main reasons for indoor tanning were perceived increased attractiveness, self‐confidence, sunburn protection, peer influence and treatment of skin conditions (Suppa et al., 2019). A recent meta‐analysis of 25 randomized controlled trials found that previous interventions for indoor tanning only had a negligible effect (*d* = .08; Sheeran et al., 2020) and therefore were inadequate. An exploration of the 20 techniques included in the interventions to reduce indoor tanning found only ‘promoting alternatives to tanning’ was effective (Sheeran et al., 2020), suggesting that lack of adequate alternatives may be a reason for use. Moreover, most of these studies were conducted in the United States and on university students (Sheeran et al., 2020), so it may not be applicable to other populations. Furthermore, whilst quantitative data can provide an overview of potential reasons for use, qualitative methods are better suited to providing a more comprehensive, nuanced understanding of behaviours and beliefs (Sutton & Austin, 2015).

The COM‐B offers a theoretical framework to explain behaviour (this is part of the Behaviour Change Wheel—a method for intervention design; Michie et al., 2011). The framework comprises three components (capability, opportunity and motivation) that can influence whether an individual continues, reduces or changes a behaviour such as indoor tanning (Michie et al., 2011). Capability is both physical (e.g., having the skills and stamina to perform the behaviour) and psychological (e.g., having the knowledge and cognitive skills to perform the behaviour). Opportunity relates to both the physical (e.g., sufficient time and necessary resources) and social (e.g., the interpersonal/ cultural influences such as others performing that behaviour) opportunity to perform a behaviour. Motivation includes reflective motivation (i.e., conscious planning/ evaluation related to the behaviour, such as having the desire to perform the behaviour) and automatic motivation (i.e., performing the behaviour without conscious planning and evaluation, e.g., habitual behaviour). Describing the reasons behind indoor tanning in terms of the COM‐B framework would allow us to determine relevant targets for intervention. Extant research suggests that interventions should target reflective motivation (e.g., to address the desire to tan to increase self‐confidence/ perceived attractiveness and to address erroneous beliefs; i.e., tanning gives protection from sunburn), physical opportunity (to provide adequate alternatives to treat skin conditions and increase attractiveness/ self‐confidence) and social opportunity (to address peer influence).

A number of qualitative studies have elicited the views/ experiences of indoor tanners. This represents a rich source of evidence on the reasons behind indoor tanning, but these data have not been formally consolidated. Therefore, this study aimed to systematically review/ synthesize the qualitative literature, utilizing the COM‐B as a framework, to identify the reasons why people use indoor tanning devices.

## METHOD

A systematic review, conducted in line with published recommendations (Moher et al., 2009), was used to identify relevant studies that used qualitative methods to explore peoples' reasons for/ experiences of indoor tanning. The review was registered with Prospero (CRD42019147911). The original protocol was amended in response to peer reviewers' suggestion to include additional databases. Also, we originally planned a meta‐ethnography but ultimately conducted a thematic/framework analysis to synthesize data using COM‐B.

### Search strategy

A systematic search was conducted in June 2019 and updated in March 2022, using PsycINFO, PubMed, Web of Knowledge and Cumulative Index to Nursing and Allied Health Literature databases. Grey literature was identified by conducting searches in OpenGrey and Trove. Search terms were developed using synonyms for indoor tanning based on a recently published systematic review and meta‐analysis (Rodriguez‐Acevedo et al., 2019) combined with terms to identify the application of qualitative methods (Table 1). Multifield search builders were used to combine keywords in accordance with the SPIDER (Sample, Phenomenon of Interest, Design, Evaluation, Research type) framework; this framework is designed specifically to identify qualitative studies, as opposed to other frameworks (e.g., PICO) that focus on quantitative research (Cooke et al., 2012).

**TABLE 1:** SPIDER framework with keywords for each search term

SPIDER reference	Search terms
Sample	Women OR men OR adults OR sunbed users OR purposive sampl$ OR theor$ sample$
Phenomenon of Interest	Indoor‐tanning OR sunbed OR tanning bed OR tanning booth OR tanning salon OR solarium OR solaria OR sunlamp OR artificial tanning OR UV tanning OR non‐solar ultraviolet radiation OR non‐solar UV radiation OR nonsolar ultraviolet radiation
Design	Questionnaire$ OR survey$ OR interview$ OR focus group$ OR case stud$ OR observ$ OR participant observ$ OR ethno$ OR phenomonolog$ OR grounded theor$ OR content analys$ OR constant compar$ method$ OR discourse analy$ OR verb$ protocol$ mixed method
Evaluation	View* OR experience* OR opinion* OR attitude* OR perce* OR belie* OR feel* OR know* OR understand*
Research type	Qualitative research OR qualitative‐study$

*Note:* $ represents truncation. Combined [S AND P of I] AND [(D or E) AND R].

### Eligibility criteria

Included studies investigated why people use indoor tanning devices. All studies had to (a) include qualitative methods for both data collection and analysis and (b) were in English. Studies that included subsamples (e.g., adults who tan in natural sunlight only; non‐users of indoor tanning devices) were included if the views of indoor tanners or ex‐users were reported separately. No restrictions were set for current or past indoor tanning use, date or quality score. Studies that did not investigate people's reasons for indoor tanning devices, that were not qualitative, that were not published in English or whose full‐text version was not retrievable were excluded.

### Study selection

**FIGURE 1:**
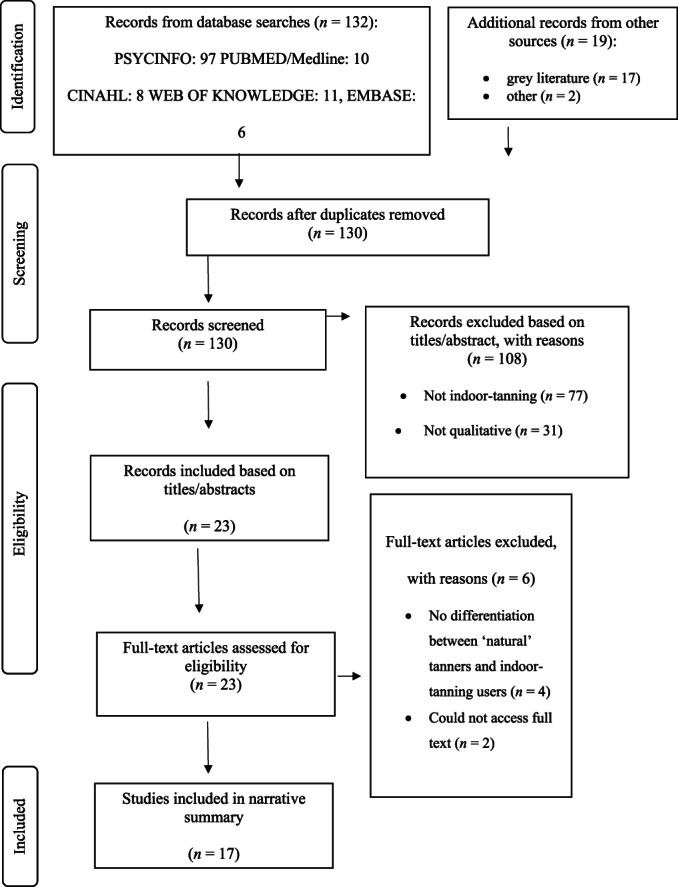
Diagram summarizing the study identification process

Search results were entered into EndNote x7 and duplicates removed. A total of 108 titles and abstracts were screened independently for eligibility against the criteria by two researchers (ME and SL); disagreement was resolved through discussion. Reasons for exclusion were recorded (Moher et al., 2009; see Figure 1). The same process was followed for the 22 articles assessed at full text. Percentage agreement at the title and abstract stage was 93% (Cohen's K = 0.74).

### Quality assessment of studies

Two researchers (ME and SL) undertook a quality assessment for each study using a 10‐item checklist developed by the Critical Appraisal Skills Programme (CASP) that provides a systematic means of appraising the validity, content and usefulness of reporting of results from qualitative studies (CASP, 2018). The use of the checklist indicated a high level of quality in all included studies, and no studies were excluded on the basis of quality (see Table S1).

### Data synthesis

Findings from each qualitative study were synthesized in a narrative summary using two processes (Hannes & Lockwood, 2011). The first was an iterative process akin to the thematic analysis of primary qualitative data, in which key overarching themes of relevance were identified (Ritchie & Spencer, 1984). The second was a framework analysis using COM‐B components. Firstly, three researchers (ME, SL and TE) familiarized themselves with the published reports of included studies. Two researchers (ME and SL) independently extracted key data from each study (e.g., year/place of study, sample characteristics and method).

In the thematic analysis process, two researchers (ME and SL) independently coded each paper and developed themes from this coding. Spreadsheets were used by each researcher to identify preliminary themes to explain indoor tanning by drawing on concepts presented in the studies.

In the framework analysis process, one researcher (TE) extracted data from each paper that reflected COM‐B components, which were then reviewed by a second researcher (SL). All themes from both processes were agreed by discussion between the three researchers (ME, SL and TE) (see Table 2 for a description of each COM item).

**TABLE 2:** COM descriptions

COM item	Description
Reflective motivation	‘Conscious planning and evaluations (beliefs about what is good and bad) e.g., I have the desire to)’
Automatic motivation	‘Doing something without thinking of having to consciously remember (e.g., “is something I do before I realise I'm doing it”)’
Physical opportunity	‘The environment provides the opportunity to engage in the activity concerned (e.g., the sufficient time, the necessary materials, reminders)’
Social opportunity	‘Interpersonal influences, social cues and cultural‐norms provide the opportunity to engage in the activity concerned (e.g., other people tanning)’
Physical capability	‘Having the physical skill, strength or stamina to engage in the activity concerned. (e.g., I have sufficient physical stamina, I can overcome disability, I have sufficient physical skills)’
Psychological capability	‘Knowledge and/or psychological strength or stamina to engage in the necessary thought processes for the activity concerned (e.g., having the knowledge, cognitive and interpersonal skills, having the ability to engage in the appropriate memory, attention and decision‐making processes)’

*Note:* All descriptions are taken from the COM‐B questionnaire (Keyworth et al., 2020).

Data were synthesized by reporting each of the COM components present—this was done for both engaging in indoor tanning and reducing/quitting indoor tanning. Within those COM categories, subthemes were discussed (many of which cut across several COM categories).

The review was reported using the PRISMA checklist (see Table S2).

## RESULTS

A total of 17 articles reporting 16 studies were included in the meta‐synthesis (see Table 3). One study's findings were reported in two articles (Gordon et al., 2016; Hay et al., 2016).

**TABLE 3:** Study characteristics

Authors (year)	Country	Type	Sample size	Range of ages of participants (mean)	Gender of participants
Vannini and McCright (2004)	USA/Canada	Interviews	40	18–52 years	Male (42%) and female (58%), tanners
Murray and Turner (2004)	UK	Interviews	18	18–32 years	Male (50%) and female (50%), tanners
Boynton and Oxlad (2011)	Australia	Focus groups	27	18–26 years (19.50)	Female (100%), tanners and non‐tanners
Lazovich et al. (2013)	USA	Focus groups	215	NA	Male (33%) and female (67%), tanners and non‐tanners
Banerjee et al. (2014)	USA	Interviews	14	(25.65)	Female (100%), tanners
Lake et al. (2014)	UK	Focus groups	69	15–18 years	Female (100%), tanners
Hay et al. (2016)	USA	Interviews	44	NA	Female (100%), tanners and non‐tanners
Gordon et al. (2016)	USA	Interviews	44	NA	Female (100%), tanners and non‐tanners
Rodgers et al. (2016)	USA	Focus groups	18	NA	Female (100%), tanners
Taylor et al. (2017)	UK	Interviews	25	NA	Male (36%) and Female (64%), tanners and non‐tanners
Kirk and Greenfield (2017)	UK	Interviews	15	NA	Male (27%) and Female (73%), tanners
Stapleton and Crabtree (2017)	USA	Key informant study	1	22 years	Female (100%), tanners
Buchanan Lunsford et al. (2018)	USA	Focus groups	159	NA	Male (54%) and female (46%), tanners and non‐tanners
Glanz et al. (2018)	USA	Interviews	40	18–25 years old	Female (100%), tanners
Taylor et al. (2018)	UK (online)	Discourse analysis	NA	NA	NA
Bowers and Moyer (2019)	USA	Open‐ended online survey items	312	18–47 years (20.72)	Female (100%), tanners
Lyons et al. (2021)	UK	Focus group and interviews	21	19–58 years	Male (14%) and Female (86%), tanners
Vannini and McCright (2004)	USA/ Canada	Interviews	40	18–52 years	Male (42%) and female (58%), tanners
Murray and Turner (2004)	UK	Interviews	18	18–32 years	Male (50%) and female (50%), tanners
Boynton and Oxlad (2011)	Australia	Focus groups	27	18–26 years (19.50)	Female (100%), tanners and non‐tanners
Lazovich et al. (2013)	USA	Focus groups	215	NA	Male (33%) and female (67%), tanners and non‐tanners
Banerjee et al. (2014)	USA	Interviews	14	(25.65)	Female (100%), tanners
Lake et al. (2014)	UK	Focus groups	69	15–18 years	Female (100%), tanners
Hay et al. (2016)	USA	Interviews	44	NA	Female (100%), tanners and non‐tanners
Gordon et al. (2016)	USA	Interviews	44	NA	Female (100%), tanners and non‐tanners
Rodgers et al. (2016)	USA	Focus groups	18	NA	Female (100%), tanners
Taylor et al. (2017)	UK	Interviews	25	NA	Male (36%) and Female (64%), tanners and non‐tanners
Kirk and Greenfield (2017)	UK	Interviews	15	NA	Male (27%) and Female (73%), tanners
Stapleton and Crabtree (2017)	USA	Key informant study	1	22 years	Female (100%), tanners
Buchanan Lunsford et al. (2018)	USA	Focus groups	159	NA	Male (54%) and female (46%), tanners and non‐tanners
Glanz et al. (2018)	USA	Interviews	40	18–25 years old	Female (100%), tanners
Taylor et al. (2018)	UK (online)	Discourse analysis	NA	NA	NA
Bowers and Moyer (2019)	USA	Open‐ended online survey items	312	18–47 years (20.72)	Female (100%), tanners
Lyons et al. (2021)	UK	Focus group and interviews	21	19–58 years	Male (14%) and Female (86%), tanners

### Study characteristics

Seventy‐six per cent of articles (*n* = 13) were published within the last five years with the earliest paper dating from 2004. Eight studies involved individual interviews (Banerjee et al., 2014; Glanz et al., 2018; Gordon et al., 2016; Hay et al., 2016; Kirk & Greenfield, 2017; Murray & Turner, 2004; Taylor et al., 2017; Vannini & McCright, 2004), and five used focus groups (Boynton & Oxlad, 2011; Buchanan Lunsford et al., 2018; Lake et al., 2014; Lazovich et al., 2013; Rodgers et al., 2016). One study involved both focus groups and interviews (Lyons et al., 2021). One paper reported a ‘key informant study’ using multiple interviews with one person (Stapleton & Crabtree, 2017), and another used discourse analysis of online texts (Taylor et al., 2018). One paper reporting results from an online survey was included because responses to two open‐ended questions had been subjected to thematic analysis (Bowers & Moyer, 2019).

Most included studies were conducted exclusively in the United States (*n* = 9). An additional study recruited participants from both the United States and Canada. The remaining studies were conducted in the United Kingdom (*n* = 6) and Australia (*n* = 1; Table 3). The majority of participants in the studies were White. Buchanan Lunsford et al. sought views of Black and Hispanic individuals around skin cancer and discovered only rare instances of indoor tanning by their participants (Buchanan Lunsford et al., 2018). Nine studies had recruited exclusively female samples with 7 including both male and female participants. The online study (Taylor et al., 2018) did not report gender of participants. As indoor tanning is predominately practised by females, there were infrequent examples of a male perspective; the majority of analyses were informed by female participants' experiences.

The appraisal indicated that qualitative methods had been applied appropriately. However, a consideration of the relationship between researcher and participants was only explicitly reported in 5 of 17 (29%) articles. Between 70% and 100% of reporting criteria were satisfactorily met by each article (see Table S1 for details).

### Explanatory factors

Five of 6 categories of the COM‐B framework were apparent in the data: reflective and automatic motivation, social and physical opportunity, and psychological capability. There was evidence that all five components influenced the likelihood of an individual engaging in indoor tanning, with all but automatic motivation also influencing quitting/reducing indoor tanning. The components are presented in the analysis in order of importance. There was no evidence of physical capability facilitating either engaging in or reducing/quitting indoor tanning.

**FIGURE 2:**
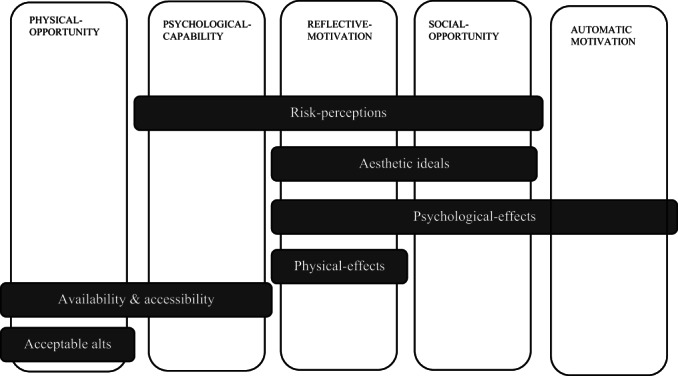
Relationship between COM factors and cross‐cutting themes

There were five subthemes identified by the thematic analysis: aesthetic values, physical effects, psychological effects, risk perceptions and acceptable alternatives. One additional theme was identified during the framework analysis: availability/accessibility. All, except acceptable alternatives and physical effects, cut across multiple COM‐B categories. See Table 4 for illustrative quotes and Figure 2 for how the themes and categories fit together.

**TABLE 4:** Main themes with supporting evidence

COM‐B	Subtheme	Engage in indoor‐tanning	Quitting/reducing tanning
Reflective motivation	Aesthetic values	We found one underlying motive for artificial tanning: the enhancement of one's appearance. Informants clearly linked body image with self‐esteem. With no exceptions, our informants told us that tanned skin improves their looks in different ways… (author interpretation) Vannini and McCright (2004) p. 319	I started to cut back after I went three times in a row and started to peel on my face [which] wasn't very attractive. I also was a lot darker than I was hoping for. (participant quote) Bowers and Moyer (2019) p. 350
			I'm not as, uh… too particular about my appearance as I was when I was younger, when I used them more frequently. (participant quote) Banerjee et al. (2014) p. 212
‘Conscious planning and evaluations (beliefs about what is good and bad) e.g., I have the desire to)’ (Taken from the COM‐B questionnaire—Keyworth et al., 2020)		My reasons in the past have been feelings of insecurity about how pale I am. Being pale in this society is not a preferred look. (participant quote) Bowers and Moyer (2019) p. 349	The main thing that would stop me is if pale became completely in. I think that's the only thing that would change my mind… (participant quote) Boynton and Oxlad, (2011) p. 976
	Physical‐effects	I needed a base tan so I would not get burned in Mexico (participant quote) Bowers and Moyer (2019) p. 349	Well, I found a mole on my left breast that I know has not been seeing the daylight, and, I had to have it removed. And I'm lucky enough that it was benign, but, um, that really opened my eyes that… you know, that could only have been caused by the tanning bed. (participant quote) Banerjee et al. (2014) p. 212
		During winter months I get seasonal depression so I use the tanning beds for vitamin D. (participant quote) Bowers and Moyer (2019) p. 349	
	Psychological‐effects	‘If I haven't been on a sunbed for a while, like when I'm trying to save money, then I just don't feel as well, as healthy. I get colds and stuff. I start to feel down and get very tense. I just don't have the willpower to stop for long’ (participant quote) Murray and Turner (2004) p. 76	‘I never really enjoyed it very much. Um, I just wasn't very comfortable with them’. (participant quote) Banerjee et al. (2014) p. 212
	Risk‐perceptions	But just about everyone seemed to downplay this risk. As one young man said: ‘What can you do these days that does not cause cancer?’ Much of this risk‐taking behavior was explained to us as a form of ‘getting the best out of life’ and ‘doing your body a little bad and a little good at the same time’. (author interpretation/participant quote) Vannini and McCright (2004) p. 326	Now, obviously, my attitude has changed. I always said that if they came out and proved that there was a link to skin cancer and tanning, that I would stop, and when they did sort of start coming out with those studies, I had stopped. I even had a membership that I was going to every once in a while, and once the studies started coming up, I just stopped and I just let the money go. (participant quote) Hay et al. (2016) p. 1266
		Maybe but I know a few people who regularly use them for years. My mum's friend who uses sunbeds, and salon owner where I go have flawless, unwrinkled skin. There is a lot of scare tactics in the media surrounding sunbeds so I would rather trust what I see with my own eyes. Also if all else fails there's always Botox (participant quote) Taylor et al. (2018) p. 523	I have thought about it*…*.my fear of cancer outweighs the aesthetic benefit of having a tan. (participant quote) Bowers and Moyer (2019) p. 346
		If you've got skin cancer you can get over it quick. (participant quote) Lake et al. (2014) p. 60	
Social‐opportunity	Aesthetic‐values	I don't know, I think boys probably go for you a bit more if you have a bit of colour (participant quote) Lake et al. (2014) p. 58	
‘Interpersonal influences, social cues and cultural‐norms provide the opportunity to engage in the activity concerned (e.g., other people tanning)’ (Taken from the COM‐B questionnaire—Keyworth et al., 2020)	Psychological‐effects	Psychological‐benefits were also mentioned, including feeling more accepted by peers, feeling more confident, and experiencing mood improvement. (author interpretation) Glanz et al. (2018) p. 296	I think maybe I grew out of it a little bit? It's that possibility, you know… after I got away from campus and college and listen to what everybody's doing, it was kind of like, ‘You know? I don't really need to do this anymore.’ (participant quote) Banerjee et al. (2014) p. 212
			[reasons for quitting] Now, a lot of my friends don't tan. … I'm the only one, so it's not necessary for me to do it. (participant quote) Glanz et al. (2018) p. 298
	Risk‐perceptions	Most of our respondents had collected information about the side effects of tanning from a variety of sources, including salon workers, popular magazine articles, Internet articles, television, and their friends. (author interpretation) Vannini and McCright (2004) p. 326	Um, well, I got a lot of… I work at a hospital, and got a lot of flack for usin’ them as we refer to them—‘The Cancer Tube’. And so, I decided (chuckles) to stop doin’ that … (participant quote) Banerjee et al. (2014) p. 212
		Finally, while discussing the barriers to quitting indoor tanning use, participants also noted the misinformation provided by tanning salons to convince customers that indoor‐tanning was safe and beneficial for them. (author interpretation) Banerjee et al. (2014) p. 214	Participants described the central role of family and friends in providing encouragement for cessation of indoor‐tanning. Accordingly, one participant noted that her mother was a nurse and frequently advised her against indoor‐tanning; therefore, she was very happy with her decision to quit. (participant quote) Banerjee et al. (2014) p. 213
Physical‐opportunity	Acceptable‐alternatives	No…Um, I've tried different lotions and everything. But I, I never really liked them, so. (participant quote) Banerjee et al. (2014) p. 216	I don't use ‘em that much, now. I might use them now for, um… for summer, like to get ready for the summer. ‘Cause again, I live in England, so it's not… there's a lot of sun and opportunity to just hang out. But, um… they've definitely improved a lot over the years. The smell's improved in the way that they… whatever it is that's interacting with their dermus, and in a way… I don't know, … splotchy… I mean, I just think there's so many, the products out there, but, I just, um…my experience with them has been good, and I've been happy with them when I want to use them, which isn't that frequently. (participant quote) Banerjee et al. (2014) p. 217
‘The environment provides the opportunity to engage in the activity concerned (e.g., the sufficient time, the necessary materials, reminders)’ (Taken from the COM‐B questionnaire—Keyworth et al., 2020)		I didn't enjoy the procedure [spray tan], I think I've done it a couple of times, but it's not something I'd do again, I'd rather just stand under a lamp and be left to daydream and you know it's like so intrusive spray tanning, I don't like it (participant quote) Lyons et al. (2021) p. 4	
	Availability & accessibility	A majority of respondents mentioned accessibility and affordability of the tanning salons as facilitators of tanning indoors (author interpretation) Glanz et al. (2018) p. 296	If it was inconvenient, like if it was not just close by. … if it was out of my way, I would just not bother (participant quote) Glanz et al. (2018) p. 297
		Yes, received phone calls offering discounts/upgrades to come back. (participant quote) Banerjee et al. (2014) p. 212	Spray tans, or stuff like that—making that more accessible or just like more widely used. (participant quote) Glanz et al. (2018) p. 297
		The gym I was a member of had indoor‐tanning facilities included in my membership, so I felt as though I may as well use them since I pay for it already. (participant quote) Bowers and Moyer (2019) p. 349	Um, well, yeah. I mean, I have kids, and so it was taking time—I was trying to do it after work and then go pick ‘em up from daycare—and it was taking away time from them, so, that was another important reason to stop’. (participant quote) Banerjee et al. (2014) p. 212
		When I go on the sunbeds I buy in bulk, it always that the sunbed's cheaper than the self tanning products, cos you can go through them quite fast and they're quite expensive (participant quote) Lyons et al. (2021) p. 4	
Automatic‐motivation	Psychological‐effects	I wish that I'd never started using one to be honest with you because I think that using a sunbed is almost like an addiction. Once you start to use them it's very hard to stop. You become used to seeing yourself with a tan and realise that you do look better. People also start to comment and say that you look nice and you just don't want to let yourself get pale again so it's very hard to stop. (participant quote) Murray & Turner p. 76	No. I did not. Um, I'd never felt addicted to tanning beds, um, I didn't have a problem stopping, just because once I made up my mind that it was the right thing to do, and that I needed to do it to be healthy, I just did it, um, and so I never desired to go back. I never had any symptoms of depression or, um… you know anything like that. (participant quote) Banerjee et al. (2014) p. 215
		There is some… something to it, where, um… it does make you feel better. And I think once… it's really, for me, it's once I START again, it's, like I just keep wanting to go back more frequently throughout the week, um… and it's hard to stop. But then once I'm stopped, I'm okay, you know. (participant quote) Banerjee et al. (2014) p. 212	
‘Doing something without thinking of having to consciously remember (e.g., “is something I do before I realise I'm doing it”)’ (Taken from the COM‐B questionnaire—Keyworth et al., 2020)		A few participants reported some psychological urges to go back for indoor‐tanning and perceived the quitting experience as somewhat difficult. One participant noted that the urge to go back to indoor‐tanning was a physical urge, and expressed it as a longing for the warmth and sensation she experienced while indoor‐tanning (therefore, we classified her urge as a psychological one). (author interpretation) Banerjee et al. (2014) p. 214	
Psychological‐capability	Risk‐perceptions	I think the one thing that I think about when you ask me about tanning salons, is just the grave amount of misinformation, and really, lying, that they do to convince customers that it's safe. And not as harmful as if you lay out in the sun; or, that it's actually good for. All these different things have ALWAYS bothered me! (Chuckles lightly)…. Um, so that's something I have run into with several different tanning salons, in the past. … I just think an awareness is… there must be a correlation between… um, education level and… ‘cause… you know, so, just being able to understand information, learn your sources…and getting lies about making decisions, I just feel like that's probably where most of my information about tanning salons come from. (participant quote) Banerjee et al. (2014) p. 213	
		‘I have a great dermatologist too… She knows I tan… She does not approve but she knows I do and so… she'll do a head to toe sweep of making sure that everything looks good and if anything is even questionable, she's like “before it turns into something worse”’ (participant quote) Rodgers et al. (2016) p. 5	
		Sometimes the positives outweigh the negatives (participant quote) Rodgers et al. (2016) p. 5	
‘Knowledge and/or psychological strength or stamina to engage in the necessary thought processes for the activity concerned (e.g., having the knowledge, cognitive and interpersonal skills, having the ability to engage in the appropriate memory, attention and decision‐making processes)’ (Taken from the COM‐B questionnaire—Keyworth et al., 2020)		The risk may increase by 70% by using them, but if the risk is only 1/20,000 to begin with, that makes it only 1/10,000 which is still microscopic and not really any different. (participant quote) Taylor et al. (2018) p. 528	
	Availability/accessibility		Um, I don't remember them calling or sending me emails, but I do remember when… when I tried to cancel? I mean, like, when I walked in to cancel? And I remember feeling a little bit nervous about going IN to cancel? But they DO try to convince you to stay, like, they start…start tellin’ you about new offers. Like, ‘Oh, we can reduce it by this much, or we can give you this new offer, or we can…’ you know? They tried to… they tried really hard to get you to stay. I mean, it's, it's hard to cancel. Um, and I was… had to go in there with a bunch of… I mean, because I… I have a hard time with that, and I KNEW that they were gonna try to do that. So I walked in there knowing, like, ‘Okay, I'm deciding I'm already gonna stop. So, no matter what they say to me, like, I've already made my decisions’. And no matter how hard they try you know, ‘I'm… I'm done’. (participant quote) Banerjee et al. (2014) p. 212

### Reflective motivation

The main driver of indoor tanning identified in the qualitative studies was reflective motivation—why people desire to perform a behaviour. There were four subthemes in reflective motivation: aesthetic values, physical effects, psychological effects and risk perceptions.

### Aesthetic values

The key motivation to engage in indoor tanning was related to individuals' aesthetic values (Banerjee et al., 2014; Buchanan Lunsford et al., 2018; Lake et al., 2014; Murray & Turner, 2004; Stapleton & Crabtree, 2017; Taylor et al., 2017; Lyons et al., 2021). Tanned skin was perceived as aesthetically desirable and helped people feel more attractive (Bowers & Moyer, 2019; Glanz et al., 2018; Rodgers et al., 2016). Tanning was also said to cover scars, help people feel slimmer and highlight muscle tone (Kirk & Greenfield, 2017; Vannini & McCright, 2004). Upcoming social events (e.g., parties, holidays) acted as a catalyst for indoor tanning, as people were more motivated to look attractive (Bowers & Moyer, 2019; Glanz et al., 2018; Lyons et al., 2021; Vannini & McCright, 2004). There is a perception amongst users that tanned skin connotes healthiness and represents wealth, ambition and a successful lifestyle (Vannini & McCright, 2004), in addition to increasing confidence and self‐esteem (Lyons et al., 2021).

Aesthetic concerns such as premature ageing or skin peeling were also often cited as an impetus for quitting (Banerjee et al., 2014; Bowers & Moyer, 2019; Buchanan Lunsford et al., 2018; Glanz et al., 2018; Hay et al., 2016; Kirk & Greenfield, 2017; Murray & Turner, 2004; Rodgers et al., 2016; Taylor et al., 2017; Vannini & McCright, 2004).

I started to cut back after I went three times in a row and started to peel on my face [which] wasn't very attractive. I also was a lot darker than I was hoping for. (Participant quote) Bowers and Moyer (2019) p. 350.

### Physical effects

Studies also cited perceived physical health benefits as a reason for engaging in indoor tanning. Some believed that tanning cured dermatological complaints (Bowers & Moyer, 2019; Lake et al., 2014; Rodgers et al., 2016; Taylor et al., 2017, 2018). Some believed that sunburn and the associated health consequences could be prevented by acquiring a tan through indoor tanning ahead of vacations (Banerjee et al., 2014; Bowers & Moyer, 2019; Glanz et al., 2018; Lyons et al., 2021; Rodgers et al., 2016; Taylor et al., 2018).

I needed a base tan so I would not get burned in Mexico. (Participant quote) Bowers and Moyer (2019) p. 349.

Another commonly reported perceived benefit was that indoor tans were a source of vitamin D (Bowers & Moyer, 2019; Lazovich et al., 2013; Lyons et al., 2021; Murray & Turner, 2004) and could reduce physical illness symptoms (Lake et al., 2014). Indeed, it was noted in one review that many users tended to highlight health aspects to avoid saying they tanned due to aesthetic reasons (Taylor et al., 2017). However, there was some evidence that when physical benefits were overshadowed by physical harm such as a mole that changed, participants were motivated to quit tanning (Banerjee et al., 2014).

### Psychological effects

Users reported that indoor tanning contributes to an improved sense of well‐being (Bowers & Moyer, 2019; Glanz et al., 2018; Hay et al., 2016; Lake et al., 2014; Lazovich et al., 2013; Murray & Turner, 2004; Rodgers et al., 2016; Vannini & McCright, 2004). Well‐being was related to enjoying the tranquillity of tanning or ‘me time’, (Lake et al., 2014), social aspects (i.e., enjoying the rapport with service providers; Stapleton & Crabtree, 2017) and the pleasant physical sensation of tanning (Vannini & McCright, 2004). Two studies identified participants who discussed how indoor tanning improved mental health conditions such as seasonal affective disorder (SAD; Bowers & Moyer, 2019; Vannini & McCright, 2004). A number of studies described how indoor tanning resulted in increased confidence due to perceived improvements in appearance (Glanz et al., 2018; Kirk & Greenfield, 2017; Lake et al., 2014; Lazovich et al., 2013; Lyons et al., 2021; Murray & Turner, 2004). Alternatively, **t**here was some evidence that those who realized they did not enjoy the process subsequently quit indoor tanning (Banerjee et al., 2014; Glanz et al., 2018).

A few participants noted that they never really enjoyed indoor‐tanning in the first place, and so were highly motivated to quit. (Author interpretation) Banerjee et al. (2014) p. 214.

### Risk perceptions

Some users downplayed the risk by considering that the risk was acceptable, for example, tanning was just one of many risky behaviours (Gordon et al., 2016; Lyons et al., 2021; Taylor et al., 2017, 2018); that the media overstated the risks (Lake et al., 2014); or in a minority of cases that the potential effects of skin cancer were insignificant (Lake et al., 2014).

If you've got skin cancer you can get over it quick.(Participant quote) Lake et al. (2014) p. 60.

But just about everyone seemed to downplay this risk. As one young man said: “What can you do these days that does not cause cancer?” Much of this risk‐taking behavior was explained to us as a form of “getting the best out of life” and “doing your body a little bad and a little good at the same time.” (Author interpretation) Vannini and McCright (2004) p. 326.

Optimistic bias was evident in some studies where people used downward social comparison (i.e., they compared the extent of their usage to others) or cited use of precautionary measures (e.g., wearing eye goggles or applying lotions) to downplay their personal risk (Rodgers et al., 2016). Some studies showed that tanners temporally discounted the risk, that is, saw the consequences of indoor tanning as distal rather than proximal (Rodgers et al., 2016). The downplaying of known risks was present even in people who had witnessed friends' and relatives' skin cancer experiences (Boynton & Oxlad, 2011). Alternatively, when participants started to accept the risks that indoor tanning posed, this led to quitting (Bowers & Moyer, 2019; Hay et al., 2016).

Now, obviously, my attitude has changed. I always said that if they came out and proved that there was a link to skin cancer and tanning, that I would stop, and when they did sort of start coming out with those studies, I had stopped. I even had a membership that I was going to every once in a while, and once the studies started coming up, I just stopped and I just let the money go. (Participant quote) Hay et al. (2016) p. 1266.

### Social opportunity

Social opportunity also influenced indoor tanning behaviours and can be summarized under three of the previous subthemes (aesthetic values, psychological effects and risk perceptions).

### Aesthetic values

The influence of social opportunity to engage in indoor tanning in relation to aesthetic values was acknowledged in a number of studies (Bowers & Moyer, 2019; Glanz et al., 2018; Kirk & Greenfield, 2017; Lake et al., 2014; Vannini & McCright, 2004). Young female indoor tanners indicated that aesthetic values were informed by their peers (Lake et al., 2014), whilst others noted celebrity influence, as active tanning was promoted as a desirable and acceptable practice (Kirk & Greenfield, 2017; Lake et al., 2014). One study found that women were under more pressure to look tanned than men (Lyons et al., 2021). Others reported that they were more attractive to others when tanned (Lake et al., 2014).

I don't know, I think boys probably go for you a bit more if you have a bit of colour (Participant quote) Lake et al. (2014) p. 58.

However, there were no reports of peers changing the value that users placed on the aesthetics of tanning or influencing their quitting behaviour, despite reporting a societal shift towards looking more ‘natural’ in recent years (Lyons et al., 2021).

### Psychological effects

Some individuals tanned in order to fit in with their peers and not ‘be the odd one out’ and in some instances were actively reminded to tan by their peers (Buchanan Lunsford et al., 2018; Glanz et al., 2018; Lake et al., 2014; Murray & Turner, 2004; Rodgers et al., 2016).

Psychological benefits were also mentioned, including feeling more accepted by peers, feeling more confident, and experiencing mood improvement. (Author interpretation) Glanz et al. (2018) p. 3.

As a corollary, individuals were likely to quit tanning when peer influence had been removed (Banerjee et al., 2014; Glanz et al., 2018).

Now, a lot of my friends don't tan … I'm the only one, so it's not necessary for me to do it. (Participant quote) Glanz et al. (2018) p.298.

### Risk perceptions

Many people were exposed to health risk information from sources that approved of indoor tanning—these were not from unbiased credible sources (e.g., magazines; Banerjee et al., 2014; Vannini & McCright, 2004).

Most of our respondents had collected information about the side effects of tanning from a variety of sources, including salon workers, popular magazine articles, Internet articles, television, and their friends. (Author interpretation) Vannini and McCright (2004) p. 326.

Meanwhile, some social influences emphasized the risk that led to quitting behaviour (Banerjee et al., 2014).

Um, well, I got a lot of… I work at a hospital, and got a lot of flack for usin’ them as we refer to them—“The Cancer Tube.” And so, I decided (chuckles) to stop doin’ that. (Participant quote) Banerjee et al. (2014) p. 212.

### Physical opportunity

There were two subthemes in physical opportunity—acceptable alternatives and availability/accessibility. Both of the subthemes were related to engaging in and reducing/quitting indoor tanning.

### Acceptable alternatives

The intention to engage in indoor tanning was attributed to a lack of acceptable alternatives. Alternative methods of tanning without radiation, involving topical application of sprays and lotions in salons or at home (also termed ‘spray’ or ‘fake’ tans), were considered unacceptable by some in terms of aesthetic quality (e.g., orange colour, uneven coverage; Lyons et al., 2021) or because of undesirable side effects (e.g., residue; Banerjee et al., 2014). Sunbathing was inconvenient (e.g., time‐consuming) and that the results from sunbathing were not as good as indoor tanning.

No…Um, I've tried different lotions and everything. But I, I never really liked them, so. (Participant quote) Banerjee et al. (2014) p. 216.

With a sunbed you are in control and it's much quicker [than sunbathing] and it's at your convenience. (Participant quote) Lake et al. (2014) p. 59.

There were some advocates for alternatives to indoor tanning products. However, they only used them infrequently to prepare for specific events or the summer (Banerjee et al., 2014). A minority who found them acceptable used them to maintain a tan after having quit indoor tanning (Banerjee et al., 2014). However, the participants did not indicate that the acceptability of alternatives was their reason for quitting.

### Availability/accessibility

Physical opportunity influenced indoor tanning due to salon availability and affordability (Banerjee et al., 2014; Glanz et al., 2018; Lyons et al., 2021).

A majority of respondents mentioned accessibility and affordability of the tanning salons as facilitators of tanning indoors. (Author interpretation) Glanz et al. (2018) p. 296.

When I go on the sunbeds I buy in bulk, it always seems that the sunbed's cheaper than the self tanning products, cos you can go through them quite fast and they're quite expensive. (Participant quote) Lyons et al. (2021) p. 4.

Some people quit tanning due to lack of time (Banerjee et al., 2014); others indicated they would quit if the salons were no longer conveniently located (Glanz et al., 2018) or that they would use alternatives if they were more available (Glanz et al., 2018).

Spray tans, or stuff like that—making that more accessible or just like more widely used. (Participant quote) Glanz et al. (2018) p. 297.

### Automatic motivation

Automatic motivation had only one subtheme, psychological effects, that was related to both engaging in and quitting indoor tanning.

### Psychological effects

Some participants talked about indoor tanning as a longing or addiction (an example of automatic behaviour) because of the psychological benefits—one participant expressed it as ‘a longing for the warmth and sensation’ (author interpretation; Banerjee et al., 2014 p.214).

There is some… something to it, where, um… it does make you feel better. And I think once… it's really, for me, it's once I START again, it's, like I just keep wanting to go back more frequently throughout the week, um… and it's hard to stop. But then once I'm stopped, I'm okay, you know. (Participant quote) Banerjee et al. (2014) p. 212.

People who did not perceive their indoor tanning as addictive found it easier to quit and did not experience psychological harm (Banerjee et al., 2014).

No. I did not. Um, I'd never felt addicted to tanning beds, um, I didn't have a problem stopping, just because once I made up my mind that it was the right thing to do, and that I needed to do it to be healthy, I just did it, um, and so I never desired to go back. I never had any symptoms of depression or, um… you know anything like that. (Participant quote) Banerjee et al. (2014) p. 215.

### Psychological capability

Psychological capability was related to risk perceptions for engaging in the behaviour and availability/accessibility for reducing/quitting tanning.

### Risk perceptions

Some people were aware of the cognitive processes (a component of psychological capability) that was sometimes unavailable to make adequate decisions about risk when faced with misinformation from salons (Banerjee et al., 2014). Others thought they were using good decision‐making processes to balance the risks (Rodgers et al., 2016; Taylor et al., 2018).

I think the one thing that I think about when you ask me about tanning salons, is just the grave amount of misinformation, and really, lying, that they do to convince customers that it's safe. … there must be a correlation between… um, education level and… ‘cause… you know, so, just being able to understand information, learn your sources…and getting lies about making decisions, I just feel like that's probably where most of my information about tanning salons come from. (Participant quote) Banerjee et al. (2014) p. 213.

The risk may increase by 70% by using them, but if the risk is only 1/20,000 to begin with, that makes it only 1/10,000 which is still microscopic and not really any different. (Participant quote) Taylor et al. (2018) p. 528.

### Availability/accessibility

When or after quitting, people had to use their interpersonal skills (a key component of psychological capability) to overcome the pressure tactics used by the salons to make their product affordable and attractive (Banerjee et al., 2014). Quitting may require less interpersonal skills in the future, as salons move to digitally managed memberships.

I remember feeling a little bit nervous about going IN to cancel? But they DO try to convince you to stay, like, they start…start tellin’ you about new offers. Like, “Oh, we can reduce it by this much, or we can give you this new offer, or we can…” you know? They tried to… they tried really hard to get you to stay. I mean, it's, it's hard to cancel. (Participant quote) Banerjee et al. (2014) p. 212.

## DISCUSSION

This study has collated the qualitative evidence to understand reasons for engaging in and reducing/quitting indoor tanning. The COM‐B provided a theoretical framework (Michie et al., 2011), to inform intervention content aimed at preventing harm from indoor tanning. Evidence is presented for the influence of individual COM‐B components on indoor tanning behaviours, and is summarized in re‐occurring subthemes.

From the synthesized evidence, we can see those influences on engaging in tanning behaviour are from reflective motivation (i.e., desiring to have an attractive appearance, better skin, improved confidence/well‐being, the belief that it is not harmful to health), social opportunity (the influence of peers/media on the norm of a tanned appearance, the psychological benefits of fitting in with tanned peers and the culture of downplaying of the risk by tanning salons), physical opportunity (the lack of acceptability of alternatives, the convenient location of salons, the affordability of tanning), automatic motivation (satisfying the addictive psychological properties) and psychological capability (the misinformation from salons affecting decision‐making processes, tanners balancing the risks). The influences on reducing/quitting tanning were reflective motivation (tanning not meeting aesthetic needs, reduction in aesthetic needs, physical harm rather than benefits, not receiving psychological benefits, acknowledging the risk), social opportunity (reduction in tanning needed to fit in with peers, influences of others on risk perceptions), physical opportunity (improvement of alternatives, salons no longer conveniently located, no longer have time), automatic motivation (not feeling addictive properties making quitting easier) and psychological capability (the interpersonal skills needed to manage the salon staff and resist incentives in order to quit).

This study suggests that interventions should focus on reflective motivation, in particular aesthetic values, which is similar to findings of other reviews that explored tanning in recreational and tourist settings (Rodrigues et al., 2013). Despite the popularity of a heavily tanned appearance declining in Western culture over recent decades, the aesthetic value achieved by indoor tanning remains fashionable and to many still denotes health and beauty (Hunt et al., 2012). There have been numerous studies of appearance‐based interventions that aim to reduce indoor tanning by highlighting that tanning conflicts with aesthetic desires in the long term. A systematic review published in 2018 found that appearance‐based interventions (e.g., using photo‐ageing technology to show a prematurely aged appearance should tanning continue) had a medium/large effect on tanning intentions (Persson et al., 2018). However, there were few studies of the effect of appearance‐based interventions on actual behaviour (Persson et al., 2018; Williams et al., 2013), and a meta‐analysis suggested this technique was ineffective (Sheeran et al., 2020). Moreover, attitudes towards appearance‐enhancing alternatives (e.g., using non‐solar tanning products, clothing, diet or exercise) did not predict actual tanning behaviour (Danoff‐Burg & Mosher, 2006). These studies suggest that interventions should focus on alternatives relevant to motivations to tan other than appearance. Some studies found that it was improved confidence rather than appearance itself that was the underlying factor for motivation (Lyons et al., 2021), which would suggest a focus on self‐esteem might be needed.

It is difficult to refute the perceived psychological benefits of indoor tanning, as they may be genuine (WHO, 2017); it is feasible that perceived improved appearance does improve self‐confidence. Furthermore, there is evidence that improved mood from indoor tanning may have a physiological basis (Fisher & James, 2010). However, benefits such as reducing the symptoms of SAD are erroneous, as this is optimally reduced by visible light therapy rather than indoor tanning (WHO, 2017). Nevertheless, in terms of intervention, psychological benefits could be met with alternatives such as meditation, spas and other forms of relaxation. For example, favourable attitudes towards using a preferred hobby to relax were negatively related to the use of indoor tanning devices (Danoff‐Burg & Mosher, 2006), suggesting this may be an alternative to promote in an intervention. These relaxing alternatives would need to be affordable and easily accessible to be acceptable as alternatives (Lyons et al., 2021).

People perceive indoor tanning to have low risks and even to have physical benefits, although both are inaccurate. For example, indoor tanning does not provide a protective tan that decreases sun damage nor does it generate sufficient vitamin D (Mendese & Gilchrest, 2012; WHO, 2017). Furthermore, commercial indoor tanning devices are not the optimal way of treating skin conditions and not recommended by health professionals (Mendese & Gilchrest, 2012; WHO, 2017). People are largely aware that tanning comes with some health risks, but they use various defensive cognitive strategies to downplay those risks, including downplaying the severity, and temporally discounting their personal risk despite accepting its existence (i.e., optimistic bias). It is noteworthy that most of the studies have been published after indoor tanning devices were deemed carcinogenic in 2009 (International Agency for Research on Cancer, 2012) and after the introduction of regulations aimed at curbing indoor tanning. This suggests that indoor tanners continue to tan despite prevalent health risk information that emphasizes risk severity or that they are choosing to get their information from less credible sources. Inaccurate health benefits of indoor tanning may be another form of defensive cognitive strategy, whereby indoor tanners avoid looking for credible information about perceived benefits and process the information on these benefits with a positive bias. Interventions that focus solely on providing information about health risks have limited success (Sheeran et al., 2014). Risk information may be particularly ineffective in indoor tanners as many already believe they understand the risks, and so they may disengage (Stapleton et al., 2010). However, there are ways to improve risk information interventions such as including efficacy information (e.g., increase people's perception that they can perform a healthy alternative behaviour and that these actions will reduce their risk; Sheeran et al., 2014). Furthermore, other behavioural change techniques can be used in conjunction with risk and efficacy information to obviate the need to use defensive strategies. For example, self‐affirmation (i.e., a process of reflecting on personally important values/strengths) removes the need to respond defensively by ensuring the person recognizes their value despite performing a maladaptive behaviour leading to increased acceptance of health risk and efficacy information (Epton et al., 2015; Epton & Harris, 2008). Such interventions also address psychological capability as they provide the environment to make unbiased decisions.

Interventions could focus on social opportunity as there is an influence of media on cultural norms around being tanned. This would involve a cultural shift from regarding tanned skin as attractive to celebrating natural skin colours. Cultural shifts have been achieved in other areas; smoking was once seen as glamorous but is now seen as an addictive health‐harming habit (Doll, 1998). However, this shift has been gradual over decades and has involved complex interventions including behaviour change components and policy regulations (Doll, 1998) suggesting that behavioural change interventions alone may not be sufficient. Another possibility is for interventions to target tanning to ‘fit in’ with peers. Research on normative interventions to change risky health behaviours (e.g., drinking alcohol in college students) suggests that peers have most influence (Perkins, 2002). It is possible that interventions could address these normative beliefs and behaviours, but individual interventions were found to be more effective than group interventions for indoor tanning (Sheeran et al., 2020). Another way to reduce social opportunity is through media literacy to give people the skills to understand that media (including social media) presents unrealistic ideals, thereby reducing the influence of social opportunity on indoor tanning (Cho et al., 2020).

Interventions that focus on physical opportunity would need to address the acceptability of alternatives and make indoor tanning less convenient/affordable. There is evidence that promoting alternatives is an effective method of reducing indoor tanning (Sheeran et al., 2020). Pagoto et al. (2010) also found that promoting tanning lotion (including providing free samples) decreased tanning behaviour in outdoor tanners. Legislation such as indoor tanning bans are also effective (Gordon et al., 2020).

There is some evidence that automatic motivation is a factor in engaging in and quitting tanning. There have been no interventions that have addressed the putative addictive properties of indoor tanning nor interventions that have addressed breaking the habit (e.g., through planning on what to do when tempted to indoor‐tan; Sheeran et al., 2020). Future research could explore these aspects.

### Strengths and limitations

The robust methodological approach to identify and summarize eligible qualitative studies is a key strength of this study. An additional strength lies in the multidisciplinary, international team who conducted the synthesis (a health economist, a health psychologist and a behavioural scientist, all without prior knowledge of indoor tanning). Subsequent analysis was informed with input from experts in the epidemiology of skin cancer, health behaviour change, health services research, qualitative methods, economic evaluation of complex interventions, valuation methods and the pathobiology and clinical management of skin cancers.

A potential limitation of this study is related to the homogeneity of the primary studies in terms of the years and the countries in which the studies took place. The primary studies were conducted over a period of fifteen years in only four countries, including multiple states of the United States. These findings may therefore not be fully generalizable to other populations. The reporting of research methods in each paper was of a generally high standard, although the relationship between researcher and participants was not always considered.

### Future research and implications of the review

The current synthesis shows that people use indoor tanning devices for many reasons beyond aesthetics. Current regulations of indoor tanning devices are suboptimal as people continue to indoor‐tan despite these restrictions. Even in the event of banning indoor tanning devices, behavioural change interventions are necessary to encourage current users to avoid using unregulated equipment. Although there are existing interventions that show promise, they tend to be narrow in scope (e.g., mainly focusing on health risks and other fear appeals such as ageing effects; Persson et al., 2018) and most of these ignore the reasons for indoor tanning other than aesthetics.

These findings suggest a range of potential interventions that influence capability (self‐affirmation interventions to allow capability to make unbiased decisions), opportunity (media literacy, per interventions, developing acceptable tanning alternatives, legislation to reduce usage) and motivation (e.g., improving confidence, devising acceptable alternatives for relaxation, self‐affirmation interventions to remove defensiveness to health risk information, and addressing addictive properties and breaking habits). The interventions could be tailored to the individual based on their reasons for tanning and barriers to quitting tanning. According to the Behaviour Change Wheel (a method of intervention design; Michie et al., 2011), the interventions should be addressed through a range of policy categories such as communication and marketing (reflective / automatic motivation, social opportunity), environmental and social planning (physical opportunity), fiscal measures (physical opportunity), legislation (physical opportunity) and service provision (automatic motivation; Michie et al., 2011).

In conclusion, a comprehensive explanation of why people use indoor tanning devices has been developed. This is the first review, which has sought to consolidate and synthesize the qualitative evidence base in the light of established behavioural change theory and a framework designed for intervention design. This work represents the necessary first step in the development of complex interventions to reduce harm from indoor tanning.

## AUTHOR CONTRIBUTIONS

**Martin Eden:** Conceptualization; data curation; formal analysis; investigation; methodology; visualization; writing – original draft; writing – review and editing. **Stephanie Lyons:** Conceptualization; data curation; formal analysis; investigation; methodology; validation; visualization; writing – original draft; writing – review and editing. **Paul Lorigan:** Conceptualization; funding acquisition; supervision; writing – review and editing. **Katherine Payne:** Conceptualization; funding acquisition; supervision; writing – review and editing. **Adele C Green:** Conceptualization; funding acquisition; supervision; writing – review and editing. **Tracy Epton:** Conceptualization; formal analysis; funding acquisition; investigation; methodology; supervision; validation; visualization; writing – original draft; writing – review and editing.

## CONFLICTS OF INTEREST

The authors have no conflicts of interest to declare.

## Data Availability

Data sharing is not applicable to this article as no datasets were generated or analysed during the current study.
